# Retraction: *E. adenophorum* Induces Cell Cycle and Apoptosis of Renal Cells through Mitochondrial Pathway and Caspase Activation in Saanen Goat

**DOI:** 10.1371/journal.pone.0336816

**Published:** 2025-11-17

**Authors:** The PLOS One Editors

Following the publication of this article [1], concerns have been raised about Fig 2-6. Specifically:

- There appear to be repetitive areas within or between the following panels:
  - In Fig 2B, all DAPI panels
  - In Fig 2B, the AO/EB 600g/kg and 800g/kg panels
  - In Fig 6A, the 600g/kg panel
- In Fig 2B, when levels are adjusted to visualize background of the AO/EB panels, the areas surrounding multiple cells appear to be discontinuous with adjacent areas of background.
- When the aspect ratio is altered, the following bands appear more similar than would be expected from independent results:
  - ◦ Lane 3 of the Fig 3A β-actin panel and lane 1 of the Fig 4A Bcl-2 panel.
  - ◦ Lanes 1-2 of the Fig 4A Bcl-2 panel and lanes 1-2 of the Fig 4D β-actin panel.
  - ◦ Lanes 1-3 of the Fig 5C Cytoplasm Cyt *c* panel in [1] and lanes 2-4 of the Fig 5A AMPK panel in DU145 cells in [2] (retracted in [3]).
- In Fig 5C, there appear to be vertical and horizontal discontinuities in the background of the Mitochondrion Cty *c* panel.

In addition to the above concerns, the Institutional Animal Care and Use Committee approval number cited in this article (DKY-B20100805) has also been cited in multiple other articles that PLOS is aware of by different authors, published between 2012 and 2019 in [4–12], which report different research objectives in different species of animals.

The Fig 5C Cytoplasm Cyt *c* panel in [1] appears similar to content previously published in [2] (retracted in [3]) under a CC BY-NC 3.0 license. The reused content was modified. The Fig 5C Cytoplasm Cyt *c* panel in [1] is subject to the license that applies to the original article.

The authors did not respond to queries about these concerns. In the absence of underlying data, the above issues remain unresolved.

In light of the above unresolved concerns that question the reliability and integrity of these results, the *PLOS One* Editors retract this article [1].

All authors either did not respond directly or could not be reached.
